# Overexpression of SMC4 activates TGFβ/Smad signaling and promotes aggressive phenotype in glioma cells

**DOI:** 10.1038/oncsis.2017.8

**Published:** 2017-03-13

**Authors:** L Jiang, J Zhou, D Zhong, Y Zhou, W Zhang, W Wu, Z Zhao, W Wang, W Xu, L He, Y Ma, Y Hu, W Zhang, J Li

**Affiliations:** 1 Key Laboratory of Protein Modification and Degradation, School of Basic Medical Sciences; Affiliated Cancer Hospital and Institute of Guangzhou Medical University, Guangzhou Medical University, Guangzhou, China; 2 Guangdong Province Key Laboratory of Brain Function and Disease, Department of Biochemistry, Zhongshan School of Medicine, Sun Yat-Sen University, Guangzhou, China; 3 Neurosurgical Research Institute, The First Affiliated Hospital of Guangdong Pharmaceutics University, Guangzhou, China; 4 Department of Pathology, The First Affiliated Hospital of Sun Yat-Sen University, Guangzhou, China; 5 Department of Neurosurgery, The First Affiliated Hospital of Jinan University, Guangzhou, China; 6 Department of Biochemistry, Zhongshan School of Medicine, Sun Yat-Sen University, Guangzhou, China

## Abstract

Overexpression of structural maintenance of chromosomes 4 (SMC4) has been reported to be involved in tumor cell growth, migration and invasion, and to be correlated with poor prognosis of cancer patient. However, its clinical significance and biological role in glioma remain unknown. Herein, we found that SMC4 expression at both mRNA and protein level was markedly increased in glioma cells and clinical tissues and that it correlated with poor prognosis. SMC4 overexpression markedly promoted the glioma cell proliferation rate and migration and invasive capability *in vitro* and *in vivo*, whereas SMC4 downregulation reduced it. Moreover, the transforming growth factor β (TGFβ)/Smad signaling pathway, which was activated in SMC4-transduced glioma cells and inhibited in SMC4-silenced glioma cells, contributed to SMC4-mediated glioma cell aggressiveness. Our results provide new insight into the oncofunction of SMC4 and the mechanism by which the TGFβ/Smad pathway is hyperactivated in gliomas, indicating that SMC4 is a valuable prognostic factor and a potential therapeutic target in gliomas.

## Introduction

Glioma is the most common primary malignant tumor of the central nervous system, accounting for about 27% of all primary brain tumors and 80% of primary malignant brain tumors.^1^ Histologically, the World Health Organization (WHO) classification of central nervous system tumors distinguishes astrocytomas, oligodendrogliomas and ependymomas, and assigns WHO grades I–IV with respect to the degree of malignancy.^2^ Although combined therapy of glioma, including surgery, radiotherapy, chemotherapy and photodynamic therapy, has progressed greatly, the clinical effects of these treatments and the prognosis of patients with glioma remain poor.^3^ The cumulative 1-year survival rate of patients with glioma is <30%, and patients with grade IV glioblastoma multiforme (GBM) have an overall median survival time of only 15 months.^4, 5^ A major contributing factor to this short survival time is the capability of glioma cell malignant proliferation, invasion and migration into the neighboring brain tissue, resulting in postoperative tumor residue and high recurrence rates.^6, 7, 8^ Therefore, the molecular mechanism underlying glioma aggressiveness warrants investigation.

Transforming growth factor β (TGFβ) is a multi-functional cytokine that promotes epithelial differentiation and inhibits cell growth;^9^ aberrant TGFβ signaling is often associated with more aggressive behavior of various tumor types,^10, 11, 12^ including glioma.^13^ TGFβ/Smad signaling is hyperactivated in high-grade gliomas, and promotes glioma cell proliferative, migration and invasive capability, which confers poor prognosis.^14, 15, 16, 17, 18^ Bruna *et al.*^14^ showed that the TGFβ pathway promotes proliferation by inducing platelet-derived growth factor subunit B in gliomas with an unmethylated platelet-derived growth factor subunit B gene. Wick *et al.*^15^ demonstrated that TGFβ promotes glioma cell invasion by inducing matrix metallopeptidase 2 (MMP2) expression and suppressing tissue inhibitor of metalloproteinases-2 expression. Liu *et al.*^16^ proposed that TGFβ-induced microRNA-10a/b expression promotes human glioma cell migration by targeting phosphatase and tensin homolog. These data indicate that TGFβ signaling promotes aggressiveness in glioma. Hence, exploring the molecular mechanism of hyperactivated TGFβ signaling in glioma would identify a valuable therapeutic target for this disease.

Structural maintenance of chromosomes 4 (SMC4), an SMC family member, encodes the SMC4 protein that is highly expressed in multiple tumors, suggesting an oncogenic role for SMC4 in cancer pathogenesis and progression.^19, 20, 21, 22, 23^ In hepatocellular carcinoma, SMC4 is highly expressed and correlates with tumor dedifferentiation, advanced stage and vascular invasion.^20, 21^ In colorectal cancer, elevated SMC4 expression promotes tumor cell growth rate, migration and invasion.^19, 22^ In prostate cancer, higher expression of SMC4 is significantly associated with the metastatic cascade and poor prognosis.^23^ Importantly, SMC4 knockdown via RNA interference suppresses cancer cell proliferation,^20, 21^ reducing the migration and invasive capability^23^ and the degree of malignancy, thereby improving overall survival,^19, 22^ suggesting that SMC4 may serve as a significant prognostic factor and potential therapeutic target. However, its expression and potential roles in glioma remain unknown.

In this study, we demonstrate that, patients with glioma had high SMC4 expression, which confers poor prognosis. SMC4 overexpression markedly promoted the glioma cell proliferation rate and migration and invasive capability *in vitro* and *in vivo*, and SMC4 downregulation reduced it. Moreover, the TGFβ/Smad pathway was hyperactivated and contributed to SMC4-mediated glioma cell aggressiveness. The results provide new insight into the oncofunction of SMC4 and the mechanisms by which the TGFβ/Smad pathway is hyperactivated in gliomas, indicating that SMC4 is a valuable prognostic factor and potential target in glioma drug therapy.

## Results

### High expression of *SMC4* mRNA in human glioma correlates with poor prognosis

**Figure 1:**
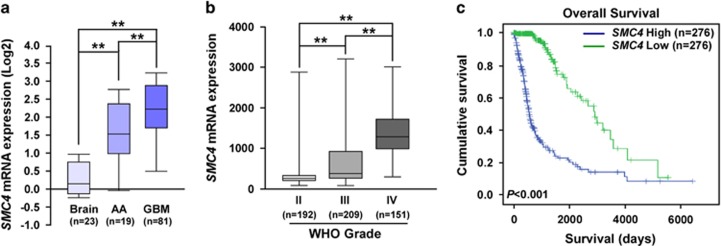
High *SMC4* mRNA expression in human glioma correlates with poor prognosis. (**a**) Quantification analysis of *SMC4* mRNA expression in normal brain tissues, anaplastic astrocytoma (AA), and GBM specimens from the Oncomine database (GSE4290 specimens). (**b**) Quantification analysis of *SMC4* mRNA expression in 522 glioma specimens of different WHO grades from TCGA. (**c**) Correlation analysis of *SMC4* mRNA expression and overall survival of 522 patients with glioma from TCGA. **P<0.01.

To explore the clinical significance of SMC4 expression in human gliomas, we first analyzed *SMC4* mRNA expression in 123 glioma specimens (19 anaplastic astrocytoma and 81 GBM) and 23 normal brain tissue samples from the Oncomine database (GSE4290 specimens). *SMC4* mRNA expression was significantly higher in anaplastic astrocytoma and in GBM than in normal brain tissues (Figure 1a), which analysis of another 552 human glioma specimens from The Cancer Genome Atlas (TCGA) confirmed (Figure 1b). The results indicate that *SMC4* mRNA expression is elevated in glioma tissues in tandem with increased WHO tumor grade. Notably, patients with higher *SMC4* mRNA expression had poorer overall survival than those with lower *SMC4* mRNA expression (Figure 1c). Survival analyses showed that high *SMC4* mRNA expression was negatively correlated with prognosis in both patients with lower-grade glioma and patients with GBM (Supplementary Figure S1) from TCGA data set, suggesting that the expression of *SMC4* mRNA is an indicator of survival in glioma.

### SMC4 protein overexpression correlates with poor prognosis in human glioma

**Figure 2:**
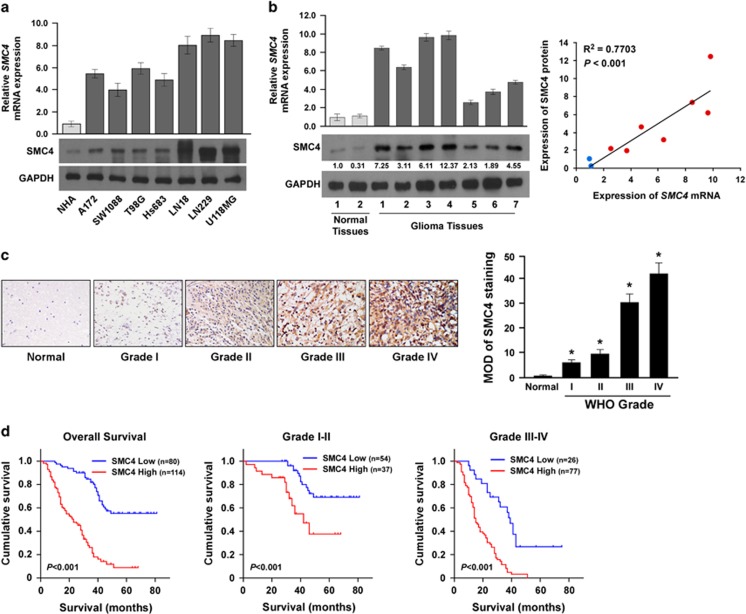
SMC4 protein overexpression correlates with poor prognosis in human glioma. (**a**) Real-time reverse transcription–PCR (RT-PCR) and western blot detection of *SMC4* mRNA and protein expression in NHA and cultured glioma cell lines (A172, SW1088, T98G, Hs683, LN18, LN229 and U118MG). (**b**) Real-time RT-PCR and western blot detection of *SMC4* mRNA and protein expression in two normal brain tissue samples and seven glioma tissue samples (left). The Spearman correlation coefficient was calculated to assess the significance of association between *SMC4* mRNA and protein expression levels (right, *P*<0.001). (**c**) IHC analysis of SMC4 protein expression in 194 glioma specimens of different WHO grades (left), magnification, × 400. Statistical quantification of the average mean optical densities (MODs) of SMC4 staining of 194 glioma specimens of different WHO grades (right). (**d**) Kaplan–Meier analysis of SMC4 expression levels in WHO grades I–IV gliomas and patient survival. *P<0.05.

To validate the above analyses, we detected *SMC4* mRNA and protein expression in normal human astrocytes (NHA), glioma cell lines and clinical specimens. Consistent with the published database, Oncomine and TCGA, the expression of not only *SMC4* mRNA but also SMC4 protein was significantly higher in the glioma cell lines than in NHA (Figure 2a). In the clinical specimens, both *SMC4* mRNA and protein were significantly elevated in the seven glioma tissue samples as compared with the two normal brain tissue samples (Figure 2b, left). Moreover, correlation analysis revealed that *SMC4* mRNA and protein expression levels correlated positively in the nine clinical specimens (Figure 2b, right), suggesting that the elevated SMC4 protein expression was probably due to amplification of the *SMC4* gene at transcriptional level.

To further evaluate the relationship between SMC4 protein expression and the clinicopathologic features of glioma, a total 194 archived, paraffin-embedded glioma specimens were analyzed by immunohistochemical (IHC) staining with an antibody against human SMC4. Among the SMC4-positive cases, 80 (41.24%) had low SMC4 expression, whereas 114 (58.76%) had high SMC4 expression (Supplementary Table S1). In interphase cells, the majority of the SMC4 condensing complex was found in the cytoplasm, therefore positive SMC4 staining was predominantly localized in the cytoplasm of glioma cells (Figure 2c, left). Furthermore, SMC4 protein expression increased markedly with human glioma WHO grade (Figure 2c, left). Quantitative IHC analysis revealed that the mean optical density of SMC4 staining in glioma cells increased significantly with the WHO grade (Figure 2c, right), suggesting that high SMC4 protein expression contributes to glioma progression.

The statistical analysis indicated that upregulated SMC4 protein was associated with WHO tumor grade (*P*<0.001) and vital status (*P*<0.001) (Supplementary Table S2). Next, we determined whether SMC4 protein upregulation correlated with poor prognosis of glioma as the disease progressed. Kaplan–Meier analysis and log-rank testing revealed that SMC4 protein expression levels in glioma specimens were inversely correlated with survival time, whether at WHO grades I–II or at WHO grades III–IV (Figure 2d). Moreover, univariate and multivariate survival analyses revealed that SMC4 expression was an independent prognostic factor of glioma (*P*<0.001) similar to the WHO grade (*P*<0.001) (Supplementary Table S3). Taken together, SMC4 protein upregulation in glioma contributes to glioma progression and correlates with poor prognosis of the disease.

### SMC4 overexpression promotes glioma cell proliferation and viability *in vitro*

**Figure 3:**
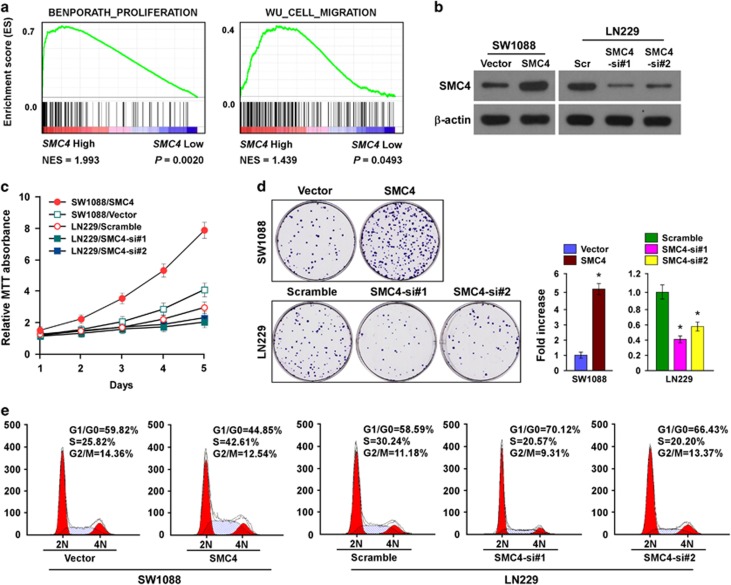
Elevated SMC4 expression promotes glioma cell proliferation and viability *in vitro*. (**a**) GSEA plot indicating a significant correlation between *SMC4* mRNA expression level and the proliferation and migration gene signatures (BENPORATH_PROLIFERATION, WU_CELL_MIGRATION). (**b**) Western blot detection of SMC4 protein expression in SW1088 and LN229 cells. (**c**) MTT assay of SW1088 and LN229 cell growth curves following SMC4 or SMC4 siRNA(s) transfection. (**d**) Representative micrographs (left) and quantification (right) of crystal violet–stained SW1088 and LN229 cell colonies following 14-day colony formation assay. (**e**) Flow cytometric analysis of cellular DNA synthesis and cell cycle progression in SW1088 and LN229 cells. Bars represent the mean±s.d. of three independent experiments. *P<0.05. Scr, scramble.

To further investigate the roles of SMC4 in glioma progression, we predicted its probable functions via bioinformatics analysis using the Gene Set Enrichment Analysis (GSEA) database. *SMC4* gene expression correlated positively with proliferation- and migration-related gene signatures in the GSEA database (Figure 3a). Therefore, we investigated the roles played by SMC4 in the proliferative and migration capability of glioma cells.

Furthermore, MTT assay was performed and revealed that SMC4 overexpression promoted SW1088 cell proliferation (Figures 3b and c). In the colony formation assay, SMC4 overexpression significantly increased the viability of the SW1088 cells, which formed more and bigger clones (Figure 3d). Conversely, silencing endogenous SMC4 in LN229 cells greatly suppressed their proliferation and viability (Figures 3b–d). The effect of SMC4 on glioma cell proliferation was confirmed in the LN18 and U118MG cell lines (Supplementary Figures S2A and B). Moreover, flow cytometry analysis was performed to investigate the effect of SMC4 on cellular DNA synthesis and cell cycle progression. SMC4 overexpression significantly decreased the percentages of cells in the G1/G0 peak and increased the percentages of cells in the S peak, whereas SMC4 knockdown significantly increased the percentages of cells in the G1/G0 peak and decreased the percentages of cells in the S peak (Figure 3e). Collectively, these data reveal that SMC4 contributes to glioma cell proliferation and viability *in vitro* by accelerating G1–S transition.

### SMC4 promotes glioma cell migration and invasive capability *in vitro*

**Figure 4:**
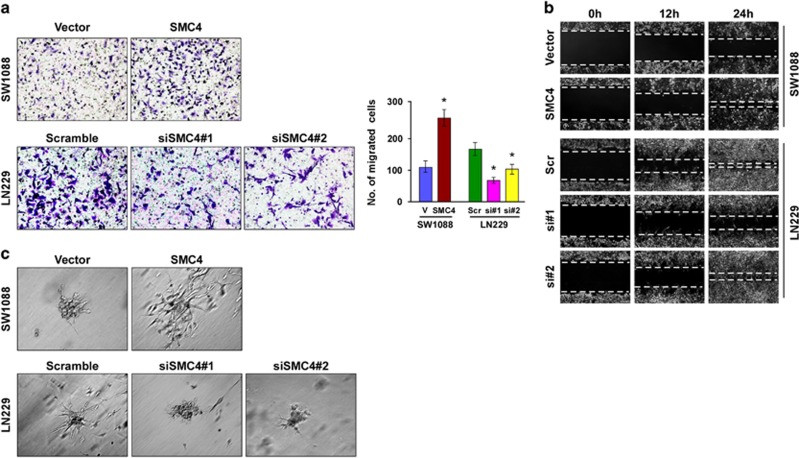
SMC4 promotes glioma cell migration and invasive capability *in vitro*. (**a**) Representative images (left, magnification, × 200) and quantification (right) of SW1088 and LN229 cell migration in the Transwell assay. The quantification of migrated cells is the mean of three independent experiments. Bars represent the mean±s.d. of three independent experiments. *P<0.05. (**b**) Wound-healing assay assessment of cell migration. (**c**) Representative micrographs of SW1088 and LN229 cells after 10-day culture in three-dimensional spheroid invasion assays, magnification, × 200. Scr, scramble.

To further understand the functions of SMC4 in glioma, we assessed the effect of SMC4 on glioma cell migration and invasion. In the Transwell assay, SMC4-overexpressing SW1088 cells showed significantly increased migration capability as compared with the vector control-transduced cells, whereas SMC4 knockdown in LN229 cells reduced their migration capability (Figure 4a). Consistently, SMC4 overexpression significantly accelerated the rate of cell migration in the wound-healing assay, whereas silencing endogenous SMC4 decreased it (Figure 4b). Similarly, the effect of SMC4 on the invasive capability of glioma cells was confirmed using the LN18 and U118MG cell lines via Transwell assay (Supplementary Figure S2C).

Furthermore, the three-dimensional spheroid invasion assay, which is considered to better mimic *in vivo* tumor invasion,^24^ revealed that SMC4-transduced SW1088 cells that had been cultured in Matrigel for 10 days displayed morphologies typical of highly aggressive invasiveness, with nearly all individual cells presenting more outward projections as opposed to the vector-transduced control cells (Figure 4c, top). By contrast, the SMC4 short hairpin RNA (shRNA)-transduced LN229 cells had immotile and spheroid morphologies (Figure 4c, bottom). These data strongly demonstrate the role of SMC4 in accelerating the migration and invasive capability of glioma cells *in vitro*.

### SMC4 accelerates glioma cell tumorigenicity *in vivo*

**Figure 5:**
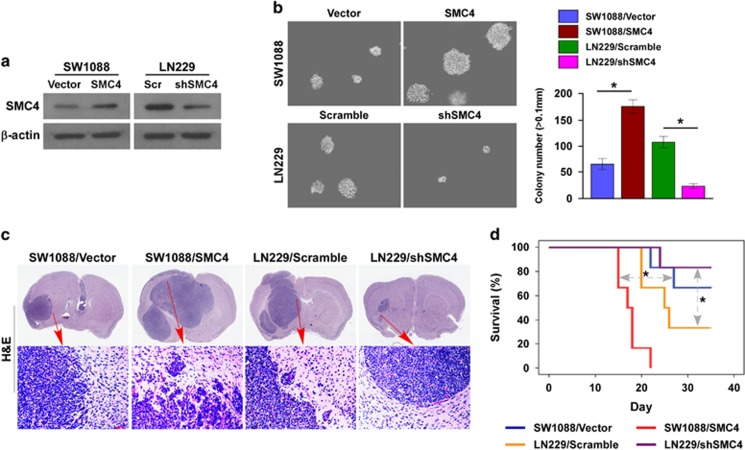
SMC4 accelerates glioma cell tumorigenicity *in vivo*. (**a**) Western blot validation of SW1088 cells stably expressing SMC4 and LN229 cells stably expressing SMC4 shRNA. (**b**) Representative micrographs (left) and quantification (right) of colonies >0.1 mm formed in the anchorage-independent growth assay. (**c**) Intracranial brain tumor xenograft model in nude mice; representative images of tumors from each group are shown. Hematoxylin–eosin (H&E, lower panel magnification, × 100) staining demonstrated that SMC4 overexpression induced the aggressive phenotype of glioma cells *in vivo*, whereas SMC4 suppression inhibited it. (**d**) Kaplan–Meier survival analysis of the mice (*n*=6 per group). Bars represent the mean±s.d. of three independent experiments. *P<0.05.

To investigate the biological role of SMC4 in glioma cell aggressiveness, we evaluated the effect of SMC4 on the tumorigenic activity of glioma cells. We established an SW1088 cell line stably expressing SMC4 and an LN229 cell line stably expressing SMC4 shRNA (Figure 5a). SMC4 overexpression significantly increased the anchorage-independent growth ability of the SW1088 cells in soft agar (Figure 5b), as indicated by the increased colony number and size, whereas silencing endogenous SMC4 decreased this tumorigenicity in the LN229 cells.

The ability of SMC4 to promote glioma progression was further examined using an *in vivo* murine model. SW1088/vector, SW1088/SMC4, LN229/scramble or LN229/shSMC4 cells were stereotactically implanted into the brains of nude mice (*n*=6 per group), and the growth morphologies of the implanted glioma tumors were examined. The SW1088/vector control cells formed mildly invasive, oval-shaped intracranial tumors (Figure 5b), with sharp edges that expanded as spheroids. In contrast, the mice that received SW1088/SMC4 cells developed highly invasive gliomas that invaded into the normal brain structures, displaying interspersed fibroblast-like structures. On the contrary, the LN229/shSMC4 cells formed noninvasive, oval-shaped intracranial tumors, with sharp edges that expanded as spheroids as compared with the LN229/scramble cells (Figure 5c). More importantly, Kaplan–Meier survival analysis demonstrated shorter survival in the mice bearing SMC4-overexpressing glioma than in the control group. In contrast, the mice bearing SMC4-inhibited tumors survived longer than the control mice (Figure 5d). The results suggest that SMC4 promotes glioma cell tumorigenicity *in vivo* along with increased proliferative and invasive capability.

### The TGFβ/Smad pathway contributes to SMC4-mediated glioma cell aggressiveness

**Figure 6:**
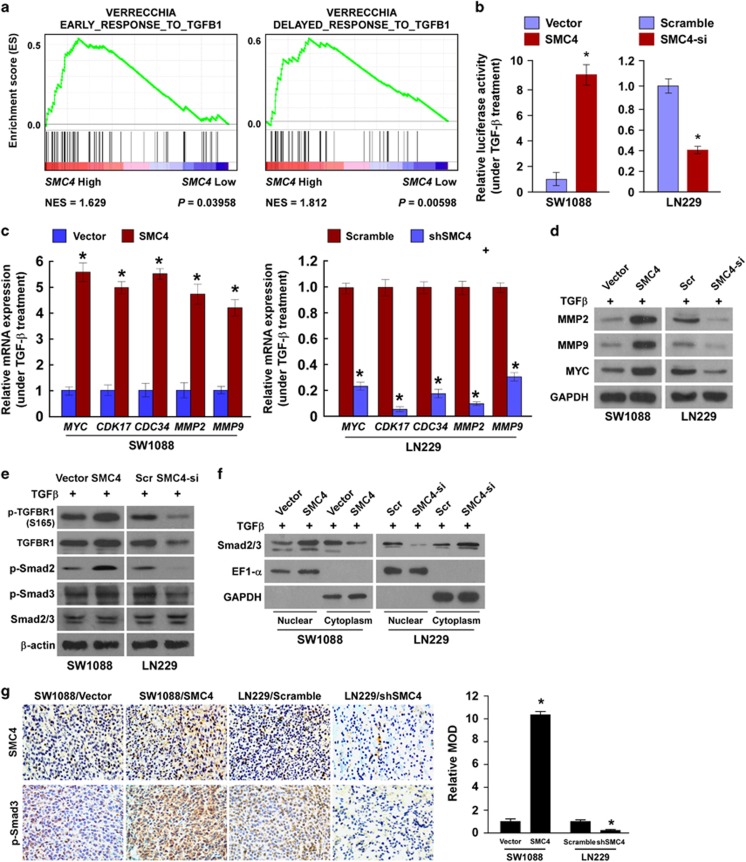
SMC4 promotes glioma cell aggressiveness by activating the TGFβ/Smad signaling pathway. (**a**) GSEA plot indicating a significant correlation between *SMC4* mRNA expression levels and early TGFβ-activated and delayed TGF-induced TGFβ gene signatures. (**b**) Luciferase-reported Smad activity in SW1088 and LN229 cells that were serum starved for 12 h before treatment with TGFβ (100 pM). (**c**) RT-PCR detection of *MYC*, *CDK17*, *CDC34*, *MMP2* and *MMP9* gene expression in SW1088 and LN229 cells that were serum starved for 12 h before treatment with TGFβ (100 pM). (**d**) Western blot detection of MMP2, MMP9 and MYC protein expression in SW1088 and LN229 cells that were serum starved for 12 h before treatment with TGFβ (100 pM). (**e**) Western blot detection of p-TGFBR1, TGFBR1, p-Smad2, p-Smad3 and Smad2/3 in the indicated cells that were serum starved for 12 h before treatment with TGFβ (100 pM). (**f**) Western blot detection of Smad2/3 expression levels in the nucleus or cytoplasm of SW1088 and LN229 cells treated with TGFβ (100 pM). (**g**) IHC staining (left) and quantification (right) of SMC4 and p-SMAD3 in the indicated tumor tissues. Magnification, × 400. Bars represent the mean±s.d. of three independent experiments. *P<0.05.

To identify the major pathways contributing to the SMC4-mediated aggressiveness of glioma cells, we carried out correlation analysis between *SMC4* expression and the possible signaling pathways in the GSEA database. *SMC4* mRNA expression correlated positively with both early TGFβ-activated and delayed TGFβ-induced gene signatures (Figure 6a), suggesting that SMC4 promotes glioma cell aggressiveness and activates the TGFβ signaling pathway.

As TGFβ/Smad pathways are central mediators of signals from the receptors for TGFβ superfamily members to the nucleus, we first assessed the effect of SMC4 modulation on Smad transcriptional activity in glioma cells by using a Smad reporter luciferase activity assay. In response to TGF-β treatment, overexpression of SMC4 significantly increased but silencing of SMC4 decreased the transcriptional activity of Smad and expression of downstream targets of TGF-β/Smad pathway (Figures 6b–d). Meanwhile, we found that the phosphorylated levels of TGFBR1, Smad2 and Smad3 and the nuclear levels of Smad2/Smad3 were markedly increased in the SMC4-overexpressing glioma cells but decreased in the SMC4-silenced cells (Figures 6e–f). However, we observed that, without TGF-β treatment, either overexpression or silencing of SMC4 did not result in any significant alterations in transcriptional activity of Smad, expression of downstream targets of TGF-β/Smad pathway and the phosphorylated levels of Smad2/Smad3 (Supplementary Figures S3A-C), suggesting that SMC4 contributes to activation of TGF-β/Smad pathway in response to exogenous TGF-β. Consistently, the expression of phosphorylated Smad3 was higher in the SMC4-overexpressing glioma tissues and lower in the SMC4-silenced tissues compared with control tumor tissues, respectively (Figure 6g), which further support the notion that SMC4 contributes to activation of TGFβ/Smad signaling.

**Figure 7:**
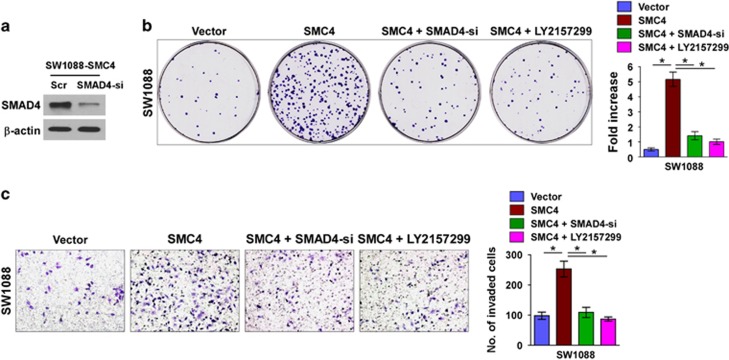
The TGFβ/Smad pathway contributes to SMC4-mediated aggressiveness of glioma cells. (**a**) Western blotting analysis of SMAD4 protein expression in SW1088 cells. (**b**) Representative micrographs (left) and quantification (right) of crystal violet-stained SW1088 cell colonies following 14-day colony formation assay. (**c**) Representative images (left, magnification, × 200) and quantification (right) of SW1088 cell invasion in the Transwell matrix penetration assay. Bars represent the mean±s.d. of three independent experiments. *P<0.05.

To further validate that SMC4-mediated glioma cell aggressiveness takes place through TGFβ activation, we blocked the TGFβ pathway in SMC4-overexpressing cells by transfecting the cells with Smad4 siRNA or the TGFβ inhibitor LY2157299 monohydrate. As shown in Figures 7a–c, inhibition of TGF-β/SMAD signaling via silencing SMAD4 or by TGF-β inhibitor Ly2157299 significantly decreased the SMC4 overexpression-induced cell proliferative and invasive capability. However, blockage of TGF-β/SMAD signaling via TGF-β inhibitor Ly2157299, even with higher dose of Ly2157299, only slightly decreased the cell proliferation and invasive capability of SMC4-silenced cells (Supplementary Figures 4A and B). These results indicate that the TGFβ/Smad pathway contributes to SMC4-mediated glioma cell aggressiveness.

## Discussion

The key finding of the present report is that SMC4 contributes to the promotion of glioma cell proliferation, migration/invasion and tumorigenicity, and activates the TGFβ/Smad signaling pathway. SMC4 was highly expressed in patients with glioma, and elevated SMC4 expression was associated with tumor progression and poor overall survival in patients with glioma, indicating that SMC4 overexpression promotes glioma cell aggressiveness and represents a novel, valuable prognostic indicator of outcome in patients with glioma.

SMC4 has clear roles in chromosome condensation and mitosis, and is required for normal S-phase progression, indicating the previously unrecognized role of SMC4 in the synchronous progression from G1 to S-phase.^25, 26^ Furthermore, SMC4 is upregulated in tumor and promotes tumor cell growth rate, migration and invasion, and is thereby involved in poor prognosis in many malignancies, including colorectal cancer, primary liver cancer, and prostate cancer, suggesting the important roles of SMC4 in tumor aggressiveness.^19, 20, 21, 22, 23^ In this study, both mRNA and protein expression of SMC4 in glioma tissues were significantly higher than that in normal brain tissues, suggesting that the elevated SMC4 protein expression was probably due to amplification of the SMC4 gene at transcriptional level. Further survival analysis revealed that elevated SMC4 correlated with poor prognosis of patients with glioma, indicating that SMC4 may serve as a valuable prognostic factor in glioma. In addition, SMC4 upregulation in glioma cells drastically increased their proliferative capability by accelerating G1–S-phase transition. SMC4 overexpression markedly promoted glioma cell migration and invasive capability *in vitro*, as well as tumorigenicity *in vivo*, which was involved in alteration of expression of the invasion-related gene MMP2, MMP9 and the proliferation marker Ki67, but SMC4 downregulation reduced it. As regulators of cell adhesion and invasion, MMP2 and MMP9 are closely involved in augmentation of the invasive capability of glioma cells and correlate with the degree of histologic malignancy and clinical outcome,^27, 28, 29, 30, 31^ whereas Ki67 indicates the proliferative ability of the tumor. We believe that SMC4 promotes glioma cell tumorigenicity *in vivo* along with the increased proliferative and invasive capability, and functions as a novel oncogene in glioma aggressiveness.

Through correlation analysis between SMC4 gene expression levels and TGFβ-related gene signatures, we predicted that SMC4 promotes glioma cell aggressiveness and activates the TGFβ/Smad pathway. The TGFβ signaling pathway is a double-edged sword in tumor development.^32^ Dual roles in cancer progression have been found for TGFβ.^33^ In metastatic prostate cancer, TGFβ suppresses cancer cell growth and motility by inducing SMADs.^34^ In hepatocellular carcinoma, TGFβ overexpression promotes tumor progression and hepatocarcinogenesis.^35, 36^ In high-grade gliomas, TGFβ functions as an oncogenic cytokine,^37^ where it is upregulated and promotes migration and invasive capability.^15, 16, 17, 18^ Consistent with the above findings, we found that TGFβ activation after SMC4 overexpression promoted glioma cell proliferative and invasive capability, whereas these enhanced capabilities were reversed after transfection with TGFβ inhibitors or Smad siRNA, suggesting that the TGFβ/Smad pathway contributes to SMC4-mediated aggressiveness of glioma cells, which may establish SMC4/TGFβ as potential therapeutic targets in gliomas.

Next, we identified the downstream target regulated by SMC4, that is, TGFβ/Smad. SMAD family proteins are molecules located downstream of the TGFβ signal transduction pathway. The Smad pathways are central mediators of signals from the receptors for TGFβ superfamily members to the nucleus. Smad2 and Smad3 are phosphorylated and activated through the formation of p-Smad2 and p-Smad3. The latter forms a transcription complex by combining with common-mediator Smads (Co-Smads), subsequently translocating to the nucleus and completing the intracellular signal transduction process.^38^ In this study, we demonstrate that SMC4 promotes glioma cell aggressiveness and activates the TGFβ/Smad pathway along with Smad2/3 phosphorylation, and enhances Smad2/3 nuclear translocation. Interestingly, immunoprecipitation assays have revealed that SMC4 interacts physically with CDK2 and CDK9,^39, 40^ which have been demonstrated to have important roles in activation of TGFβ/Smad pathway via phosphorylation of SMAD3.^41, 42^ Therefore, it is worthy to further investigate the effect of CDK9 and CDK2 on SMC4-mediated activation of TGFβ/Smad pathway.

In summary, we demonstrate that SMC4 upregulation promotes glioma cell aggressiveness, such as cell proliferation, migration/invasion and tumorigenicity. The aggressiveness-promoting role of SMC4 in gliomas is associated with the activation of Smad expression and TGFβ transactivity. Understanding the biological function of SMC4 in glioma progression will not only advance our knowledge of the mechanisms underlying glioma aggressiveness, but also establish SMC4 as a significant prognostic factor or a potential therapeutic target for treating gliomas.

## Materials and methods

### Cell culture

Primary NHA were purchased from the Sciencell Research Laboratories (Carlsbad, CA, USA) and cultured as recommended by the manufacturer. Glioma cell lines (A172, SW1088, T98G, Hs683, LN18, LN229 and U118MG) were purchased from American Type Culture Collection (ATCC, Manassas, VA, USA) and grown in the completed medium, which Dulbecco’s modified Eagle’s medium supplemented with 10% fetal bovine serum (HyClone, Logan, UT, USA) and 100 units penicillin–streptomycin, at 37 °C with 5% CO_2_ atmosphere in a humidified incubator. The completed medium supplemented with TGFβ (100 pM) was used for function experiment.

### Clinical specimens

A total of 194 archived paraffin-embedded glioma specimens were obtained from the First Affiliated Hospital of Sun Yat-Sen University and the First Affiliated Hospital of Guangdong Pharmaceutics University from 2005 to 2010, including 23 cases of pilocytic astrocytomas (grade I), 68 cases of diffuse astrocytoma (grade II), 64 cases of anaplastic astrocytoma (grade III) and 39 cases of GBM (grade IV). Another seven glioma specimens and two normal brain samples frozen in liquid nitrogen were collected for extracting mRNA and protein. The two normal brain tissues were obtained from individuals who died of traffic accidents and who were histopathologically confirmed to be free of pre-existing pathological lesions. No patients had received any antitumor treatments before biopsy. For the use of these human materials, prior consents and approval from the Institutional Research Ethics Committee were obtained. The clinical information for the patient samples was summarized in Supplementary Table S1.

### RNA extraction, reverse transcription and real-time RT–PCR

Total RNA was extracted from freshly frozen samples or cells with TRIzol reagent (Invitrogen, Carlsbad, CA, USA). Total RNA was reverse-transcribed with First Strand cDNA Synthesis Kit (Invitrogen). Real-time PCR reactions were conducted using Platinum SYBR Green qPCR SuperMix-UDG reagents (Invitrogen) on the Applied Biosystems (Foster City, CA, USA) 7500 Sequence Detection system. All reactions were done in triplicate and reactions without reverse transcriptase were used as negative controls. Human actin beta (*ACTB*) was used as the endogenous controls and the 2^−ΔΔCT^ equation was used to calculate the relative expression levels. The primers used for detecting genes expression are shown in Supplementary Table 5.

### Western blot analysis

Western blot analysis was conducted using anti-SMC4, anti-MMP2, anti-MMP9, anti-Ki67, anti-phospho-Smad2, anti-phospho-Smad3 and anti-Smad2/3 antibodies (Cell Signaling Technology, Danvers, MA, USA), anti-TGFBR1 and anti-phospho-TGFBR1(Abcam, Cambridge, MA, USA), Human glyceraldehyde3-phosphate dehydrogenase, actin beta (ACTB) or elongation factor 1 alpha (EF1-α) was used as the endogenous reference according to details.

### IHC assay

IHC staining was carried out using anti-human SMC4 antibody (Cell Signaling Technology). The degree of immunostaining of SMC4 in formalin-fixed, paraffin-embedded sections was reviewed and scored based on both the proportion of positively stained tumor cells and the intensity of staining. The proportion of tumor cells was scored as follows: 0 (no positive tumor cells), 1 (<10% positive tumor cells), 2 (10–50% positive tumor cells) and 3 (>50% positive tumor cells). The intensity of staining was graded according to the following criteria: 0 (no staining); 1 (weak staining=light yellow), 2 (moderate staining=yellow brown) and 3 (strong staining=brown). The staining index was calculated as staining intensity score proportion of positive tumor cells. Using this method of assessment, we evaluated the expression of SMC4 in glioma specimens by determining the staining index, which scores as 0, 1, 2, 3, 4, 6 and 9. Moreover, above-mentioned different cut-offs values were examined using the log-rank test. Using scores of 3, or 4 or 6 as cut-off value, the log-rank test showed that the survival time was significantly different between the low and high SMC4 expression groups (*P*<0.05). Then the median value, which score index=4, was chosen as the cut-off value. Therefore, samples with a score index⩾ 4 were determined as high expression and samples with a score index<4 were determined as low expression. The images were captured using the AxioVision Rel.4.6 computerized image analysis system (Zeiss, Oberkochen, Germany).

### Vectors, retroviral infection and transfection

pSmad-luc and control plasmid (Clontech, Mountain View, CA, USA) were used to quantitatively examine Smad activity. pMSCV/SMC4 was generated by subcloning the PCR-amplified human SMC4 coding sequence into pMSCV vector (Clontech). Human SMC4 targeting shRNA oligonucleotides sequences (RNA#1: 5′-GCAAAGAGCTCATTAGCAATG-3′ and RNA#2: 5′-GGTGGTGGAAGCAAAGTAATG-3′) were cloned to generate pSuper-retro-SMC4-RNAi(s), respectively. Transfection of plasmids was performed using the Lipofectamine 3000 reagent (Invitrogen). Retroviral production and infection were performed as described previously.^43^ Stable cell lines expressing SMC4 or SMC4 shRNA were selected for 10 days with 0.5 μg/ml puromycin 48 h after infection. In this same way, stable cell lines expressing Smad shRNA (RNAi#1, 5′-GCATGTTAAGGAAACTCAGCC-3′, and RNAi#2, 5′-GCAGCGCCTCATCAAGAAAGT-3′) were established and selected.

### MTT assay

Glioma cells were seeded at 1500 cells per well in 96-well plates after transfection. MTT assay was performed to test cell viability at 1, 2, 3, 4 and 5 days, and the absorbance was measured at 490 nm with a spectro photometric plate reader.

### Colony formation assay

Cells were plated at 500 cells per well in six-well plates after transfection, and cultured for 14 days. Colonies were fixed with methanol, stained with 0.5% crystal violet and counted under the inverted microscope.

### Flow cytometry assay

The indicated cells were harvested, washed with cold phosphate-buffered saline and processed for cell cycle analysis by using flow cytometry. Briefly, the cells were fixed in 75% ethanol and stored at −20 °C for later analysis. The fixed cells were centrifuged at 1000 r.p.m. and washed with cold phosphate-buffered saline twice. RNase A (20 mg/ml final concentration) and propidium iodide staining solution (50 mg/ml final concentration) was added to the cells and incubated for 30 min at 37 °C in the dark. Fifty thousand cells were analyzed by using a FACSC alibur instrument (BD Biosciences, Bedford, MA, USA) equipped with CellQuest 3.3 software (BD Biosciences, Bedford, MA, USA). ModFit LT 3.1 trial cell cycle analysis software was used to determine the percentage of cells in the different phases of the cell cycle.

### Transwell assay and transwell matrix penetration assay

Cells (1 × 10^4^) were plated on the top side of polycarbonate Transwell filter (without Matrigel for Transwell assay) or plated on the top side of polycarbonate Transwell filter coated with Matrigel (for Transwell matrix penetration assay) in the upper chamber of the BioCoat Invasion Chambers (BD Biosciences) and incubated at 37 °C for 22 h, followed by removal of cells inside the upper chamber with cotton swabs. Migrated and invaded cells on the lower membrane surface were fixed in 1% paraformaldehyde, stained with hematoxylin, and counted (ten random 100 × fields per well). Cell counts were expressed as the mean number of cells per field of view.

### Three-dimension spheroid invasion assay

The indicated cells (1 × 10^4^) were trypsinized and seeded on 2% Matrigel coated in 24-well plates, and medium was refreshed every other day. Cells forming a three-dimension spherical structure (spheres) were photographed at 2-day intervals for 10 days.

### Anchorage-independent growth assay

Cells (5 × 10^2^) were trypsinized and suspended in 2 ml completed medium plus 0.3% agar (Sigma, St Louis, MO, USA). The agar-cell mixture was plated on top of a bottom layer with 1% agar completed medium mixture. At 10 days, viable colonies that were larger than 0.1 mm were counted. The experiment was carried out for three times independently for each cell line.

### Intracranial brain tumor xenografts, IHC and hematoxylin–eosin staining

Indicated cells (5 × 10^5^) were stereotactically implanted into individual nude mouse brains (*n*=6 per group). The glioma-bearing mice were killed after 5 weeks, whole brains were removed and 6-μm sections were cut and subjected to IHC and hematoxylin–eosin staining. After deparaffinization, sections were IHC analyzed using an anti-SMC4 antibody (Cell Signaling Technology) or hematoxylin–eosin-stained with Mayer’s hematoxylin solution. The images were captured using the AxioVision Rel.4.6 computerized image analysis system (Zeiss).

### Luciferase reporter assay

Fifty thousand cells per well were seeded in triplicates in six-well plates and were allowed to settle for 12 h. One hundred nanograms of pSmad-luciferase plasmid or control-luciferase plasmid plus 10 ng pRL-TK renilla plasmid (Promega, Madison, WI, USA) were transfected into glioma cells by using the Lipofectamine 3000 reagent (Invitrogen). Medium was replaced after 6 h, and luciferase and renilla signals were measured 48 h after transfection by using the Dual Luciferase Reporter Assay Kit (Promega) according to a protocol provided by the manufacturer.

### Statistical analysis

The statistical tests for data analysis included Fisher’s exact test, log-rank test, χ^2^ test and Student’s two-tailed *t*-test. Univariate and multivariate statistical analysis was performed using a Cox regression model. Statistical analyses were performed using the SPSS 11.0 statistical software package (Chicago, IL, USA). The data represented mean±s.d.; *P*⩽0.05 was considered statistically significant.
