# Correction: Overexpression of SMC4 activates TGFβ/Smad signaling and promotes aggressive phenotype in glioma cells

**DOI:** 10.1038/s41389-022-00442-2

**Published:** 2022-11-18

**Authors:** L. Jiang, J. Zhou, D. Zhong, Y. Zhou, W. Zhang, W. Wu, Z. Zhao, W. Wang, W. Xu, L. He, Y. Ma, Y. Hu, W. Zhang, J. Li

**Affiliations:** 1 Key Laboratory of Protein Modification and Degradation, School of Basic Medical Sciences; Affiliated Cancer Hospital and Institute of Guangzhou Medical University, Guangzhou Medical University, Guangzhou, China; 2 Guangdong Province Key Laboratory of Brain Function and Disease, Department of Biochemistry, Zhongshan School of Medicine, Sun Yat-Sen University, Guangzhou, China; 3 Neurosurgical Research Institute, The First Affiliated Hospital of Guangdong Pharmaceutics University, Guangzhou, China; 4 Department of Pathology, The First Affiliated Hospital of Sun Yat-Sen University, Guangzhou, China; 5 Department of Neurosurgery, The First Affiliated Hospital of Jinan University, Guangzhou, China; 6 Department of Biochemistry, Zhongshan School of Medicine, Sun Yat-Sen University, Guangzhou, China

Correction to: *Oncogenesis* 10.1038/oncsis.2017.8, published online 13 March 2017

Following the publication of this article the authors noted that an incorrect image had been presented in Fig. 5c. The correct lower image of intracranial brain tumor of SW1088/Vector control mice is provided below. This correction has no effect on the conclusions of the article.

**Fig. 5:**
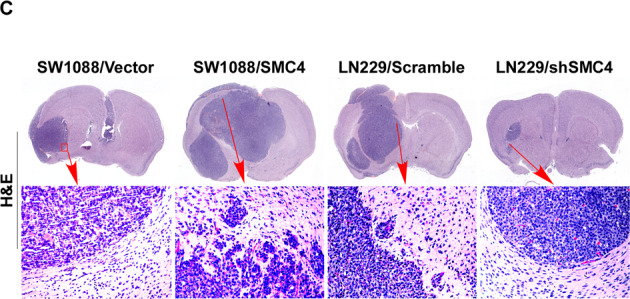
SMC4 accelerates glioma cell tumorigenicity in vivo. **c** Intracranial brain tumor xenograft model in nude mice; representative images of tumors from each group are shown. Hematoxylin–eosin (H&E, lower panel magnification, × 100) staining demonstrated that SMC4 overexpression induced the aggressive phenotype of glioma cells in vivo, whereas SMC4 suppression inhibited it.

