# Supplementary material for: Overexpression of SMC4 activates TGFβ/Smad signaling and promotes aggressive phenotype in glioma cells
Source: Oncogenesis. 2017 Mar 13;6(3):e301–. doi: 10.1038/oncsis.2017.8 (PMC5533949; doi:10.1038/oncsis.2017.8)
Supplement: Supplementary Information [file oncsis20178x1.doc]

**Supplementary Figure Legends**

**Supplementary Figure S1. *SMC4* mRNA expression is negatively correlated with prognosis in both patients with lower-grade gliomas and patients with GBM. A,** Kaplan–Meier survival curves of 401 patients with lower-grade glioma with high versus low *SMC4*-expressing tumors from TCGA. **B,** Kaplan–Meier survival curves of 543 patients with GBM with high versus low *SMC4*-expressing tumors from TCGA.

**Supplementary Figure S2. SMC4 overexpression promotes glioma cell invasive capability. A,** Western blot detection of SMC4 protein expression in LN18 and U118MG cells. **B,** Quantification of crystal violet–stained cell colonies following 14-day colony formation assay. **C,** Quantification of invaded cells in the Transwell matrix penetration assay. Bars represent the mean ± SD of three independent experiments. **P* < 0.05.

**Supplementary Figure S3. TGF-β stimulation is essential for SMC4-mediated activation of TGFβ/Smad pathway. A,** Luciferase-reported Smad activity in SW1088 and LN229 cells. **B,** RT-PCR detection of *MYC*, *CDK17*, *CDC34*, *MMP2*, and *MMP9* gene expression in SW1088 and LN229 cells. **C,** Western blot detection of p-Smad2, p-Smad3, and Smad2/3 in the indicated cells.

**Supplementary Figure S4. A,** Quantification of crystal violet–stained cell colonies formed by the indicated cells treated with Ly2157299. **B,** Quantification of invaded cells treated with Ly2157299 in the Transwell matrix penetration assay. Bars represent the mean ± SD of three independent experiments. **P* < 0.05.

**Supplementary Table S1.** **Clinicopathological characteristics of studied patients and expression of SMC4 in** **230 glioma specimens**

| Characteristics | No. of Cases | % |
| --- | --- | --- |
| **Age (years)** |  |  |
| ≤45 | 132 | 68.04 |
| >45 | 62 | 31.96 |
| **Gender** |  |  |
| Male | 128 | 65.98 |
| Female | 66 | 34.02 |
| **WHO grade** |  |  |
| I | 23 | 11.86 |
| II | 68 | 35.05 |
| III | 64 | 32.99 |
| IV | 39 | 20.10 |
| **Vital Status (at follow-up)** |  |  |
| Alive | 75 | 38.66 |
| Dead | 119 | 61.34 |
| **SMC4 expression** |  |  |
| Low expression | 80 | 41.24 |
| High expression | 114 | 58.76 |

**Supplementary Table S2.** Correlation between SMC4 expression and clinicopathological characteristics of 194 glioma specimens.

| **Characteristics** | | **SMC4** | | **Chi-square test**  ***P*-value** |
| --- | --- | --- | --- | --- |
|  |  | **Lower**  **expression** | **Higher expression** |  |
| **Gender** | Male | 54 | 74 | 0.708 |
|  | Female | 26 | 40 |  |
| **Age** | ≤ 45 | 63 | 69 | 0.007 |
|  | > 45 | 17 | 45 |  |
| **WHO grade** | I | 20 | 3 | <0.001 |
|  | II | 34 | 34 |  |
|  | III | 22 | 42 |  |
|  | IV | 4 | 35 |  |
| **Vital Status** | Alive | 50 | 25 | <0.001 |
|  | Death | 30 | 89 |  |

**Supplementary Table S3.** Univariate and multivariate analyses of various prognostic parameters in patients with glioma by Cox-regression analysis

|  | **Univariate analysis** | | | **Multivariate analysis** | | | |
| --- | --- | --- | --- | --- | --- | --- | --- |
|  | **No. patients** | ***P*** | **Regression coefficient (SE)** | ***P*** | **Relative risk** | | **95% confidence interval** |
| **Age** |  | | | | | | |
| ≤ 45 | 132 | <0.001 | 0.006 | 0.913 | 0.999 | | 0.987-1.012 |
| > 45 | 62 |  |  |  |  |  |  |
| **WHO grade** |  |  |  |  |  | |  |
| **I** | 23 | <0.001 | 0.116 | <0.001 | 2.288 | | 1.781-2.939 |
| **II** | 68 |  |  |  |  |  |  |
| **III** | 64 |  |  |  |  |  |  |
| **IV** | 39 |  |  |  |  |  |  |
| **Expression of SMC4** |  | | | | | | |
| **Low** | 80 | <0.001 | 0.222 | <0.001 | | 3.222 | 1.995-5.203 |
| **High** | 114 |  |  |  |  |  |  |

**Supplementary Table S4.** Primers used for detecting *SMC4*, *MYC*, *CDK17*, *CDC34*, *MMP2*, *MMP9* and *ACTB* genes

| **Primers** | **Sequence(5'-3')** |
| --- | --- |
| *SMC4* primer | F: GAGAAAATTCTGGGACCTTT |
|  | R: TCTGAATGTCCTTGTGTTCA |
| *MYC* primer | F: TCAAGAGGCGAACACACAAC |
|  | R: GGCCTTTTCATTGTTTTCCA |
| *CDK17* primer | F: AAGAGAAGGCTATCCCTCACAC |
|  | R: ATAGGCTCATTATCCTTGCTGC |
| *CDC34* primer | F: GACGAGGGCGATCTATACAACT |
|  | R: GAGTATGGGTAGTCGATGGGG |
| *MMP2* primer | F: ATACAGGATCATTGGTTACACACC |
|  | R: GCTGCCACGAGGAATAGG |
| *MMP9* primer | F: TTGGTCCACCTGGTTCAACT |
|  | R: ACGACGTCTTCCAGTACCGA |
| *ACTB* primer | F: GCACAGAGCCTCGCCTT |
|  | R: GTTGTCGACGA CGAGCG |
