# Supplementary figures and images for: Overexpression of SMC4 activates TGFβ/Smad signaling and promotes aggressive phenotype in glioma cells
Source: Oncogenesis. 2017 Mar 13;6(3):e301–. doi: 10.1038/oncsis.2017.8 (PMC5533949; doi:10.1038/oncsis.2017.8)

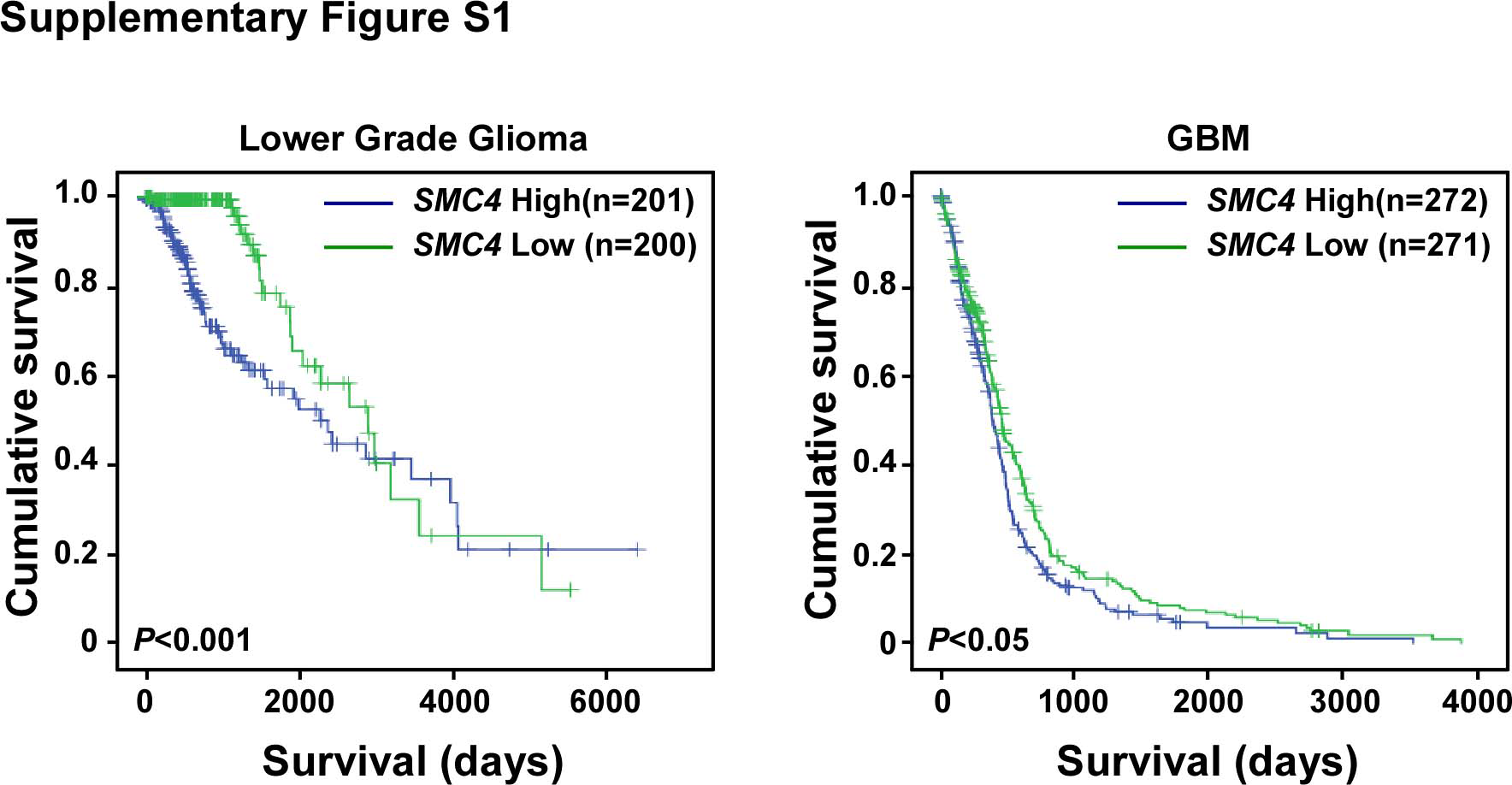

Supplement: Supplementary Figure S1 [file oncsis20178x2.tif]

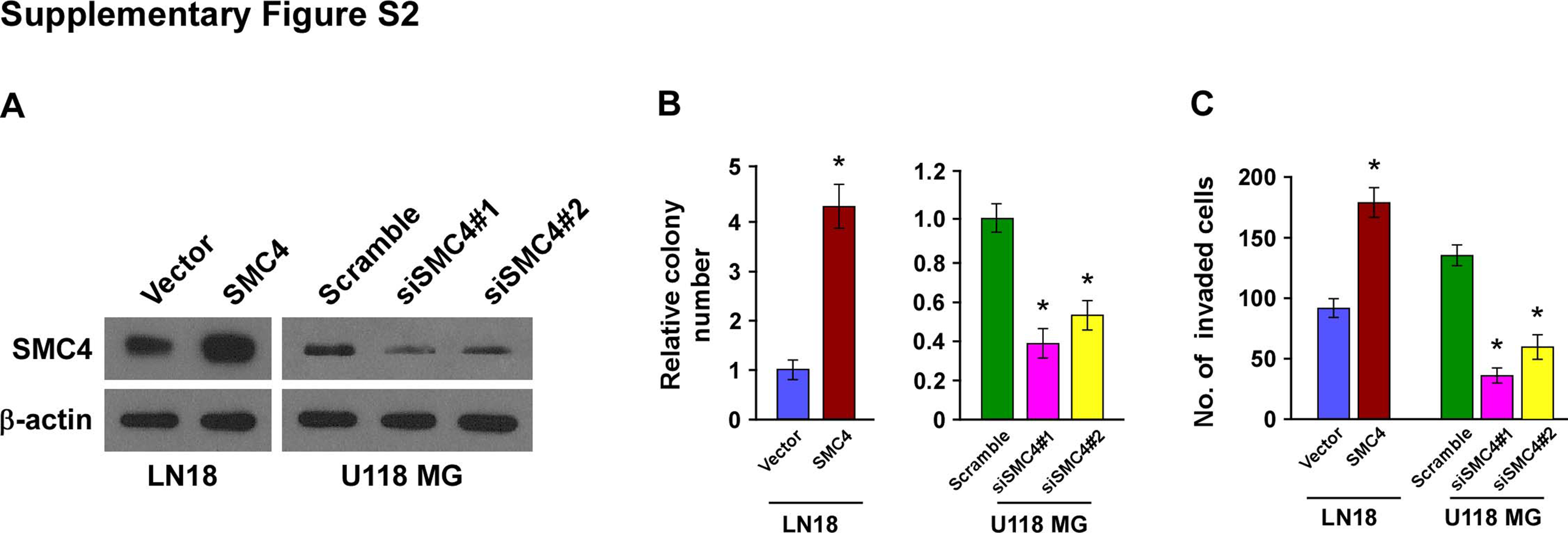

Supplement: Supplementary Figure S2 [file oncsis20178x3.tif]

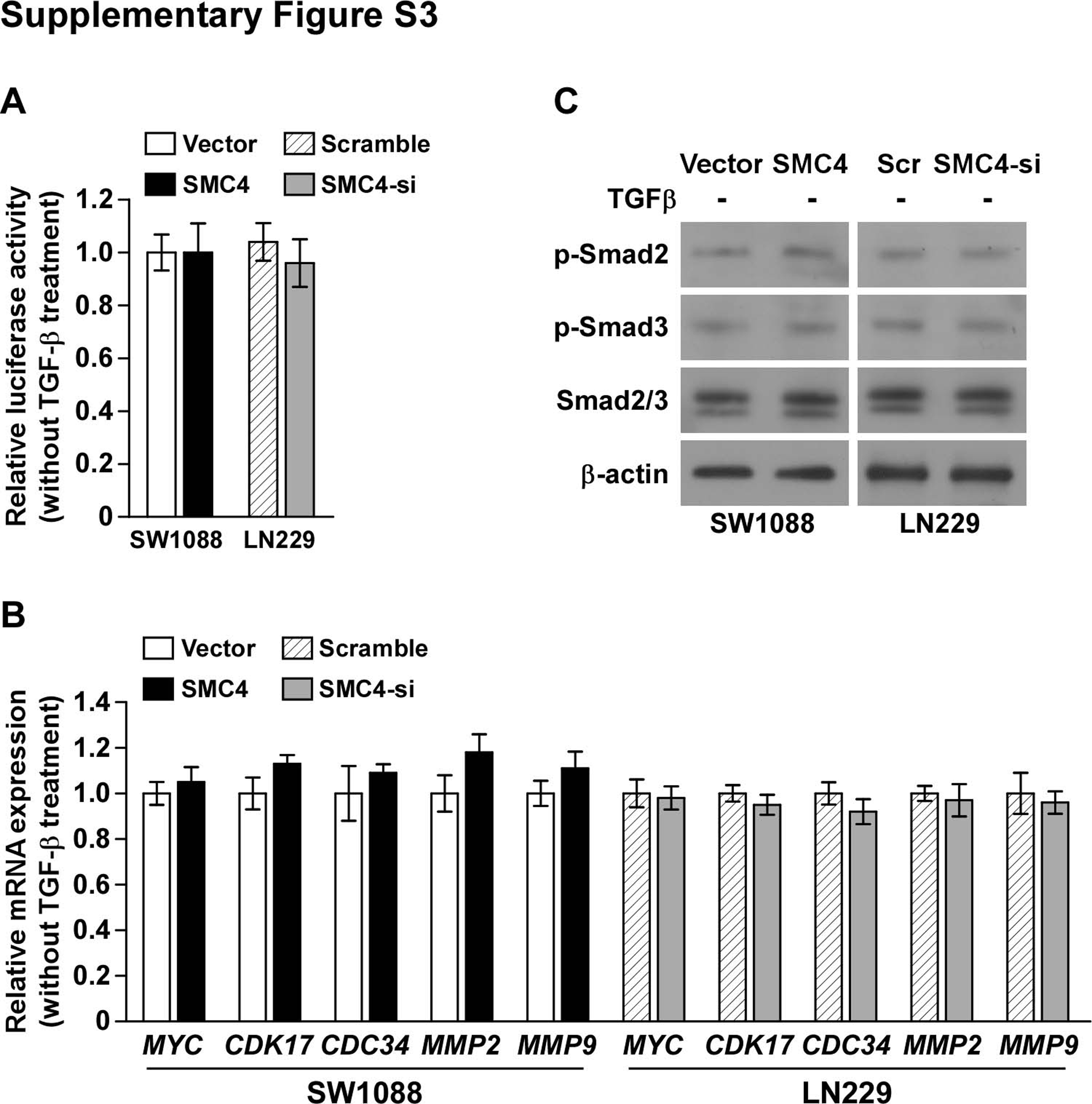

Supplement: Supplementary Figure S3 [file oncsis20178x4.tif]

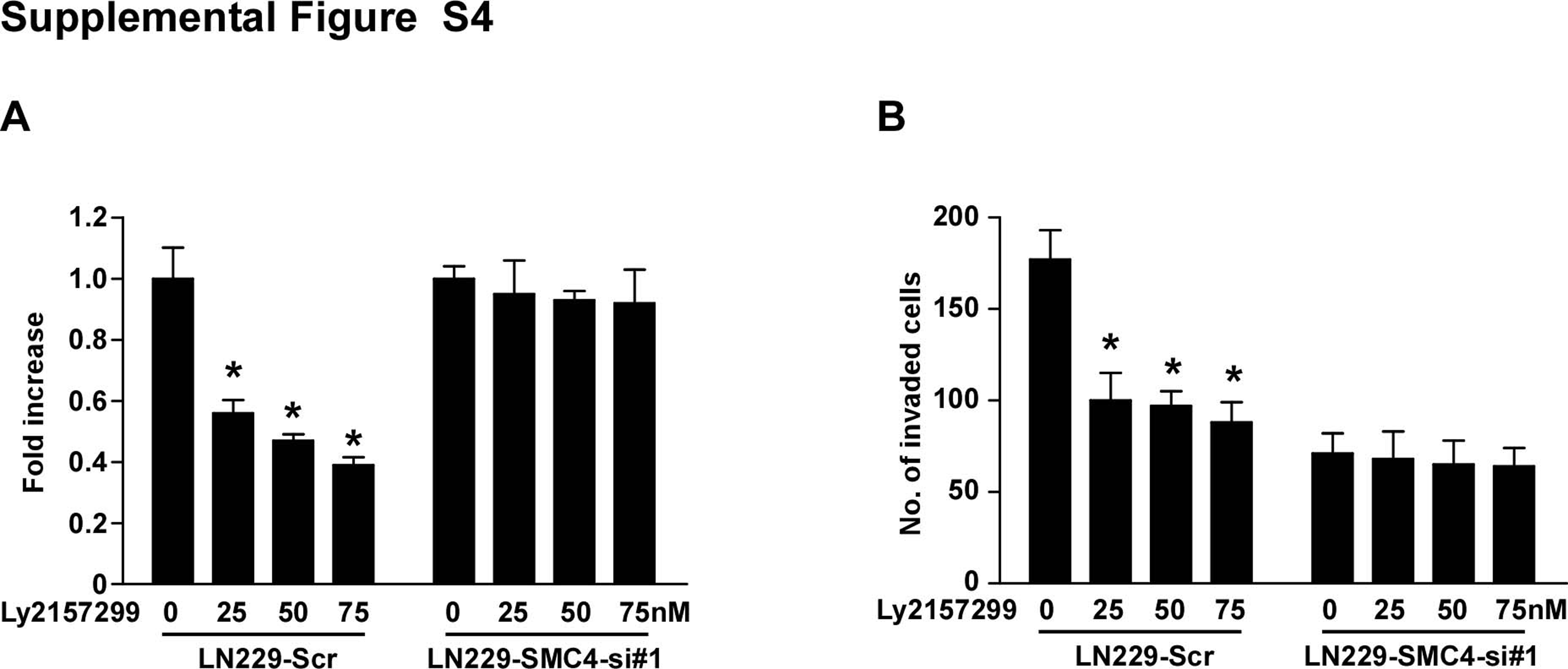

Supplement: Supplementary Figure S4 [file oncsis20178x5.tif]
